# Assessing Adventitious Matches of Non-Donors Related to True Contributors

**DOI:** 10.3390/biom15030398

**Published:** 2025-03-11

**Authors:** Kaitlin Huffman, Lilliana Moreno

**Affiliations:** DNA Support Unit, Federal Bureau of Investigation Laboratory, Quantico, VA 22135, USA; limoreno@fbi.gov

**Keywords:** probabilistic genotyping, complex mixtures, related non-donors

## Abstract

Relatives share more DNA with one another than unrelated individuals. Therefore, there is an increased risk of close relatives (e.g., siblings or parents and children of true donors) adventitiously ‘matching’ DNA mixtures to which they are not a true contributor. One such method of addressing relatives is to utilize alternative propositions within probabilistic genotyping software (e.g., STRmix^TM^). As the number of related individuals within a mixture increases, so does the potential for adventitious matches of related non-donors to the mixture due to increased allele sharing. The extent to which siblings and parents/children of true donors result in adventitious matches to mixtures in which they are non-donors is presented as well as the impact of overestimating the number of contributors (NOC) when related donors are in question.

## 1. Introduction

Related individuals can be problematic for DNA-mixture interpretation due to an increased degree of allele sharing when compared to unrelated individuals [1]. This leads to an increased risk of non-donor adventitious matching when a close relative (e.g., sibling, parent, or child) is present within a mixture [2]. It has been shown these adventitious matches are most prevalent in low-template samples with a lot of drop-out [3].

Various approaches have been developed to account for relatives, such as the use of unified LRs, which considers the presence of relatives within the population when calculating the LR. However, since the majority of the population are assumed to be unrelated (i.e., >99.99%), often, little to no impact is seen on the LRs obtained when <~10^5^ (i.e., below the ‘very strong support’ level) [4,5].

As the number of donors or relatives present within a mixture increases, so does the risk of adventitious matching. This further creates difficulty in number of contributor (NOC) determinations due to allele sharing, often resulting in underestimated NOCs, which may result in false exclusions of true donors [6]. Therefore, in this study, mixtures were analyzed according to both the experimental NOC (eNOC, i.e., the number of individuals’ DNA present in the initial mixture) and an analyst-determined NOC (aNOC, i.e., analyst manual assessment), potentially providing both best- and worst-case scenarios. The low-template nature of some mixtures (e.g., 8–16 pg) or a low donor proportion ultimately leads to an overestimated NOC using eNOC, while aNOC has the potential to be underestimated due to allele overlap. The extent to which adventitious matching of siblings and parents/children of true donors to complex 1–5-person samples occurs is presented, as well as the impact of overestimating the NOC when related donors are in question.

## 2. Materials and Methods

### 2.1. DNA Mixtures

Buccal swabs were collected from unrelated donors with informed consent and extracted using the EZ1 Advanced XL (Qiagen Sciences, Inc., Germantown, MD, USA) with the EZ1 DNA Investigator Kit (Qiagen Sciences, Inc.). Half the donor extracts were degraded via UV irradiation (180 s) by placing the extracts with open tubes in the SPECTROLINKER XL-1000 UV Crosslinker (Spectronics Corporation, Westbury, NY, USA). Extracts were quantified using the Quantifiler^TM^ Trio Kit (ThermoFisher Scientific, Carlsbad, CA, USA) and the ABI 7500 Real Time PCR System according to the manufacturer’s recommended protocols. Two- through five-person mixtures with various donor ratios were created (Table S1). Each initial mixture (e.g., Mx1, Mx11, Mx21, etc.) was diluted and amplified at 10 concentrations ranging from 8 pg to 4 ng (note: 4 ng samples were not run for five-person mixtures). Samples were amplified with the GlobalFiler^TM^ amplification kit (ThermoFisher Scientific, Carlsbad, CA, USA) at 28 cycles according to manufacturer-recommended protocols. Samples were then injected on the Applied Biosystems 3500 Genetic Analyzer using Module J6 (24 sec injections, 1.2 kV, 60 °C, NT4400). GeneMapper^TM^ v1.6 (ThermoFisher Scientific, Carlsbad, CA, USA) and FaSTR^TM^ DNA v1.1.1 (Institute of Environmental Science and Research, Auckland, NZ and Forensic Science SA, Adelaide, SA, Australia) were used for analysis. Dye-specific analytical thresholds were used with each software (Table S2). Manual NOC assessments (aNOC) were determined by considering the number of alleles, peak height balances, and apparent peaks below the AT.

### 2.2. STRmix^TM^

Each GeneMapper^TM^ v1.6-examined mixture was analyzed with STRmix^TM^ v2.7 (Institute of Environmental Science and Research, Auckland, New Zealand) according to the experimentally designed NOC (eNOC) as well as the manually determined NOC (aNOC). Two databases were created. The first database contained known donors and 202 unrelated non-donors, while a second database comprised 50 simulated siblings and 50 simulated children from each donor per mixture. Database searches were conducted (Table 1) using a Fst = 0.01 and the sub-source LRs reported. A subset of eNOC = aNOC mixtures were additionally analyzed Section 2.1 using NOC + 1 in STRmix^TM^ to evaluate the impact of overestimating the NOC.

### 2.3. FaSTR^TM^

Each sample was analyzed in FaSTR^TM^ DNA v1.1.1. All OMR and OL alleles were removed and neural network allele calls applied. Each sample was then manually reviewed to ensure artifact peaks were removed. A database matching search was conducted on both previously mentioned databases. The percentage of alleles per donor from the databases present (i.e., %matching) in each mixture was reported.

## 3. Results and Discussion

**Theorem 1.**  *Likelihood ratio when considering unrelated donors.*

(1) $$LR=\frac{POI+{\left(NOC-1\right)}_{unrelated}}{NOC_{unrelated}},$$

**Figure 1:**
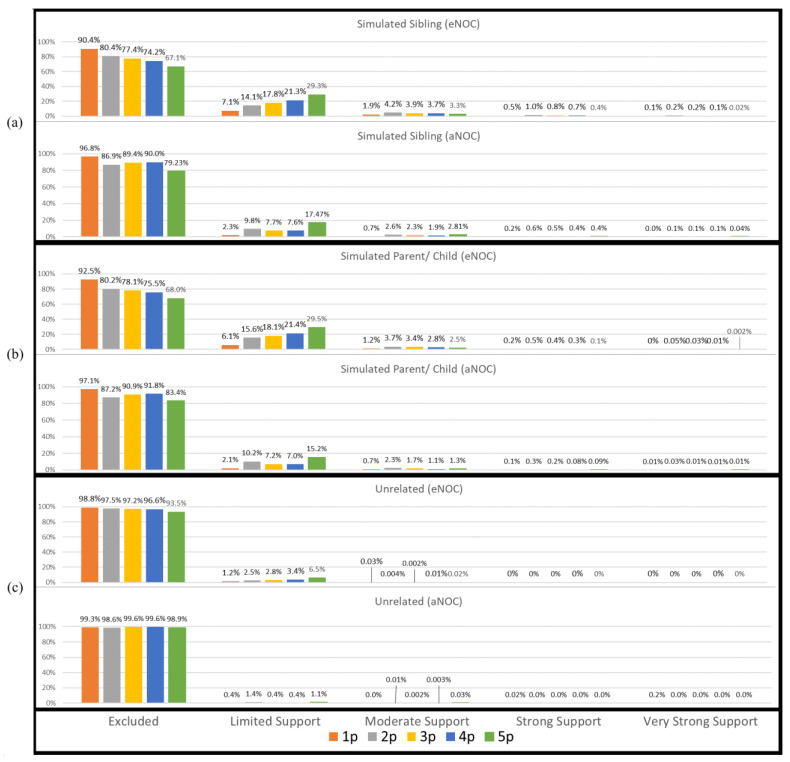
Non-donor sub-source LRs ranked according to SWGDAM verbal qualifiers. (**a**) Simulated siblings; (**b**) simulated parents/children; (**c**) unrelated. Note: excluded (LR < 1); limited support (LR 2–99); moderate support (LR 100–9999); strong support (LR 10,000–999,999); very strong support (LR ≥ 1,000,000).

Consistently with previous studies [7,8,9,10,11], there was an increased risk of true donor relatives adventitiously matching to mixtures to which they did not contribute their DNA. However, the overall rate at which this occurred was low (Figure 1). Using a traditional LR (Theorem 1), a majority of simulated relatives (e.g., siblings or parents/children) were accurately excluded as donors to the mixtures. However, there were instances in each NOC category where the simulated sibling and simulated parents/children resulted in adventitious matches with “Very Strong Support” (i.e., LR ≥ 1,000,000) (Figure 1). In this study, sub-source LRs were reported; however, if the lower HPD (highest posterior density) LRs had been reported instead, it is expected that the LRs would have been approximately one order of magnitude lower, slightly decreasing the number of adventitious matches.

**Figure 2:**
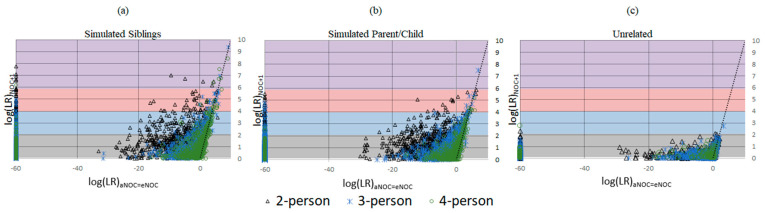
Comparison of mixture adventitious matching logs (LRs) analyzed at NOC _(aNOC = eNOC)_ and NOC _(aNOC = eNOC)_ + 1. Gray background log 0–2: ‘limited support’ for NOC + 1 samples; blue background log 2–4: ‘moderate support’ for NOC + 1 samples; pink background log 4–6: ‘strong support’ for NOC + 1 samples; purple background log ≥ 6: ‘very strong support’ for NOC + 1 samples per SWGDAM verbal qualifiers. Black triangle: two-person mixtures; blue asterisk: three-person mixture; green circle: four-person mixture. (**a**) Simulated sibling non-donors (*n* = 106 mixtures). (**b**) Simulated parent/child non-donors (*n* = 106 mixtures). (**c**) Unrelated non-donors (*n* = 582 mixtures).

Applying the eNOC (ground truth NOC based on the constructed mixture) has the effect of overestimating the NOC relative to the aNOC, allowing for higher rates of adventitious inclusions. This is particularly true for lower-template but higher-eNOC mixtures (Figure 1), where one or more ground-truth donors has dropped out enough so that the aNOC would usually be considered lower than the eNOC. Previous studies have demonstrated that overestimating the NOC by one generally results in limited impact to true donor LRs, while unrelated non-donor LRs tend to trend toward 1 [12,13,14,15]. However, underestimation of the NOC can result in excluding true donors. To assess the effect of overestimating the NOC when the POI is a relative of the true donor, a comparison of the eNOC and aNOC-generated LRs was made (Figures S1 and S2). Additionally, a subset of mixtures (*n* = 106) where the experimental NOC and apparent NOC appeared to be equal (i.e., eNOC = aNOC) were then evaluated according to the NOC _(aNOC = eNOC)_ and NOC _(aNOC = eNOC)_ + 1 to further evaluate this impact, while ensuring the initial NOC was not already elevated due to donor drop-out. Some non-donor relatives resulted in adventitious matches with ‘strong and very strong support’ (i.e., LR ≥ 10,000 and LR ≥ 1,000,000) when they were previously exclusionary (Figure 2a,b). This indicates that overestimating the NOC allows for persons with similar profiles (i.e., relatives) to be included, with the missing alleles of these non-donors probably being accounted for by drop-out (Table S3). These high adventitious matches primarily occurred with lower NOC mixtures (e.g., two-person mixtures run as three-person mixtures: Figure 2—black triangles). In accordance with previously published data, if the POI was an unrelated individual, the LRs trended toward 1 (Figure 2c). It should be noted that the NOC is more likely to be underestimated due to allele sharing [14,16,17] leading to an aNOC < eNOC. However, as shown here, overestimation, when it does occur, can be especially problematic when considering related POIs.

**Figure 3:**
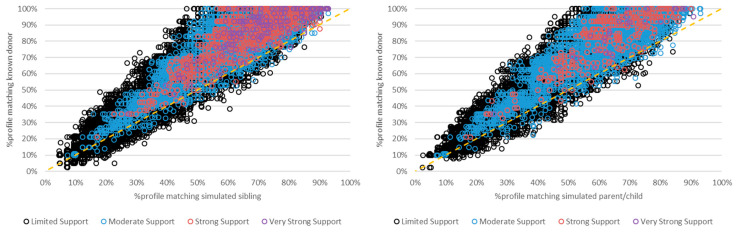
Percentage of the POI’s (i.e., known donor’s or simulated relative’s) genotype profile matching the mixture using a FaSTR^TM^ database search (2–5-person mixtures that resulted in eNOC adventitious matches for the non-donor relative). Left: simulated siblings; right: simulated parents/children. Dashed line: y = x; black circles: ‘limited support’; blue circles: ‘moderate support’; pink circles: ‘strong support’; purple circles: ‘very strong support’ per SWGDAM verbal qualifiers. Note: limited support (LR 2–99); moderate support (LR 100–9999); strong support (LR 10,000–999,999); very strong support (LR ≥ 1,000,000).

**Figure 4:**
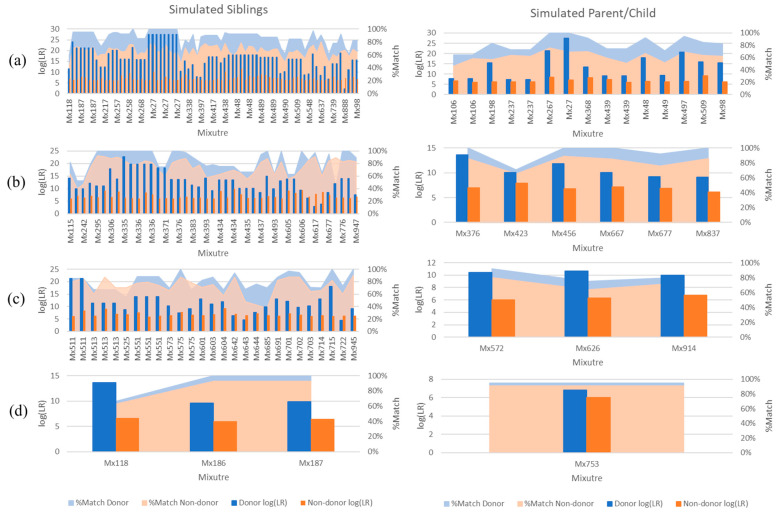
aNOC mixtures that resulted in adventitious matching for non-donor relatives with ‘very strong support’ (i.e., log (LR) > 6). Comparison of sub-source LRs (STRmix^TM^ v2.7 with GeneMapper^TM^ data) for known donors and non-donor simulated siblings; percentage of POI (i.e., known donor or simulated sibling) profile matching the mixture using a FaSTR^TM^ database search. (**a**) Two-person; (**b**) three-person; (**c**) four-person; (**d**) five-person.

FaSTR was used to determine the percentage of alleles present in each mixture for both true contributors and simulated non-donor relatives. As expected, true donors typically had a higher percentage of allele matching in each mixture than their simulated relatives (Figure 3 and Figure 4). There were instances in which non-donors had a slightly higher percentage of matching to the mixture. However, these instances mostly resulted in (eNOC) adventitious inclusions with limited to moderate support (i.e., LRs between 2 and 9999), while many were exclusionary when analyzed with aNOC. Similarly, adventitious matches resulting from related non-donors had lower or equivalent LRs in most instances compared to their true donor relatives (Figure 4 and Table S4). The maximum LR achieved for a non-donor relative (sibling aNOC = 2 p) was 2.95 × 10^11^, indicating that the extremely high LRs (e.g., LRs > ~10^15^) were always indicative of a true donor.

**Figure 5:**
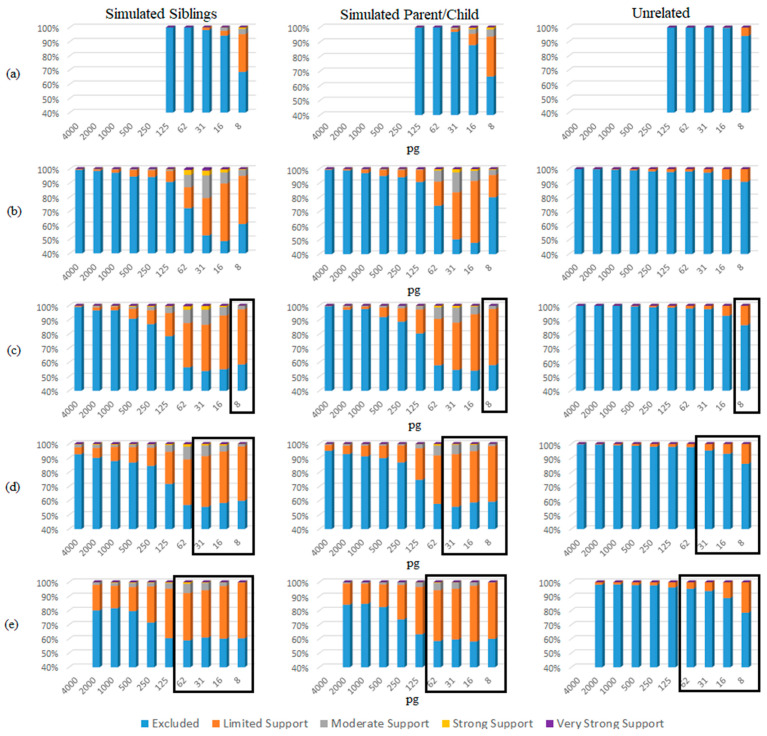
eNOC sub-source LRs organized by concentration (pg) and SWGDAM verbal qualifiers. (**a**) Single source; (**b**) two-person mixtures; (**c**) three-person mixtures; (**d**) four-person mixtures; (**e**) five-person mixtures; boxed: concentrations evaluated based on eNOC but not observed in aNOC data (see Figure 6).

**Figure 6:**
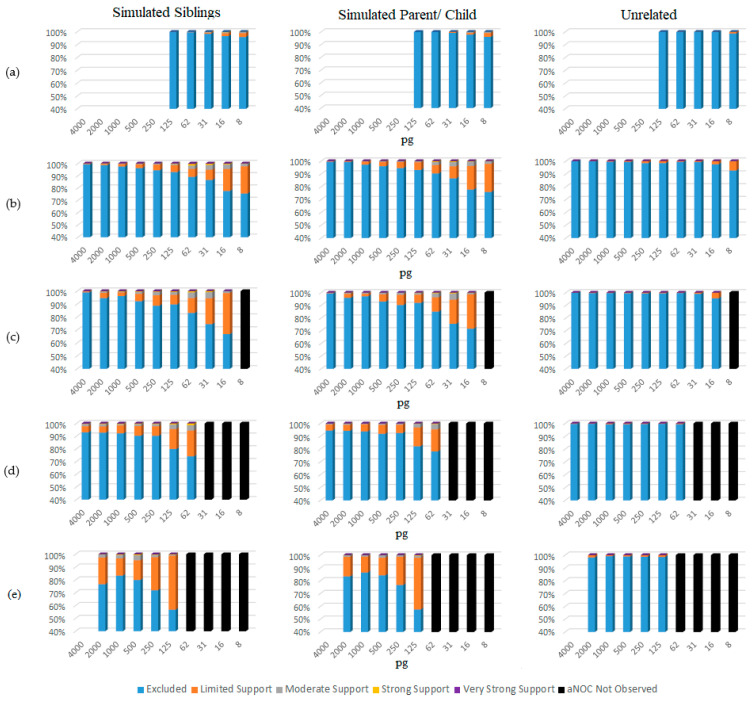
aNOC sub-source LRs organized by concentration (pg) and SWGDAM verbal qualifiers. (**a**) Single source; (**b**) two-person mixtures; (**c**) three-person mixtures; (**d**) four-person mixtures; (**e**) five-person mixtures.

The eNOC adventitious matches were more likely in low-template samples experiencing drop-out (Figure 5 and Table S5), with STRmix^TM^ often placing the non-donor as a low-template level contributor—typically either in the same donor position as the true contributor relative or in a lower position. This overestimation essentially allowed STRmix^TM^ to divide the detected donor’s alleles to enable inclusion of the non-donor via allele masking and drop-out. These adventitious matches occurred even in instances when the relative was the major donor to the mixture (more prevalent in lower NOCs (e.g., NOC = 2)), indicating that the adventitious match was explained by the non-donor being considered a minor donor with masked peaks. The aNOC-interpreted mixtures (Figure 6 and Table S6) resulted in a steady increase in adventitious matches as concentration decreased, with high aNOCs (e.g., four or five-person) not being seen at lower concentrations, as expected (Figure 5). If it is considered that diploid cells contain ~6.6 pg of DNA, this is expected. For example, aNOCs of 4 were not detected in <31 pg mixtures, as this would equate to ~1 cell per donor in an equimolar mixture. This further supports the notion that some of the eNOC adventitious matches are due to an overestimated NOC. Adventitious matches for mixtures were maximized at a concentration of ~31 and 62 pg for eNOC runs. Instead of steadily increasing with decreased concentration as seen with aNOC runs, the degree of adventitious matching started to level out or slightly decrease due to the drop-out of true-donor-shared alleles (i.e., the drop-out of the alleles the true donors shared with their non-donor relatives). This leveling out (most likely due to an overestimated NOC) corresponded to concentrations that were not observed with an aNOC.

For single-source samples, only samples with a concentration of 125 pg and below were assessed, as drop-out was not expected at higher concentrations. For these samples, adventitious matches did not occur with relatives until concentrations of <31 pg, regardless of whether the eNOC or aNOC was assessed (Figure 5 and Figure 6 and Table S5 and Table S6). However, adventitious matches of unrelated non-donors did not occur until concentrations of <16 pg (Figure 5 and Figure 6 and Table S5 and Table S6).

In many instances, adventitious matches occurred for multiple relatives in the same mixture (e.g., Mx27 Figure 4a). As most individuals do not have 50 siblings or children, the rate of adventitious matching that could occur in casework is therefore probably lower. There are some methods commonly known that may further limit these adventitious matches, such as conditioning the profile on a known donor such as a victim to reduce the mixture complexity [18] (e.g., Theorem 2). The impact of conditioning on a donor to the mixtures shown in Figure 4b–d resulted in a decrease in support for ~97% of the conditioned samples.

**Theorem 2.**  *Likelihood ratio when conditioning on a known donor.*

(2) $$LR=\frac{POI+Known\;donor+{\left(NOC-2\right)}_{unrelated}}{Known\;donor+{\left(NOC-1\right)}_{unrelated}}$$

Familial database searches were also conducted on the true donors to see what LR ranges could be expected for true donors in instances in which it was unknown if they were a true donor or the relative of a donor. In most cases, true donors resulted in familial LRs higher than those of their simulated relatives, potentially providing further support for them being true donors (Figure S3).

## 4. Conclusions

Adventitious matching of non-donor relatives occurred for both siblings and parents/children of true donors in all NOC classes tested (i.e., 1–5 persons). While overestimating the NOC when considering unrelated individuals typically has limited impact, overestimating the NOC when the POI is a potential relative can be detrimental, as non-donor relatives may result in adventitious matches with ‘strong and very strong support’ (i.e., LR > 10,000 and LR > 1,000,000) when they have previously been exclusionary. The eNOC is never known in casework samples, and the risk of an adventitious inclusion by overestimating the NOC is minimal, as the NOC is more likely to be underestimated rather than overestimated due to allele masking or the drop-out of trace donor alleles. However, rare occurrences in which the NOC is overestimated may be observed due to drop-in.

Future work assessing the degree of adventitious matching of non-donor relatives to mixtures in which two or more of their first-degree relatives are present is planned (e.g., mixtures containing multiple siblings, or parents and children) as well as further studies addressing NOC estimations.

## Figures and Tables

**Table 1:** Hd true tests (i.e., non-donors) conducted per mixture and relation type.

NOC	1	2	3	4	5
Samples	78	230	236	232	190
Simulated children	50	50	50	50	50
Simulated siblings	50	50	50	50	50
Hd true tests per relative type	3900	23,000	35,400	46,400	47,500

## Data Availability

Additional data (not already provided as part of the publication) might be made accessible upon request to the corresponding author should it comply with internal privacy, legal, and/or ethical rules and regulations.

## Supplementary Materials

The following supporting information can be downloaded at: https://www.mdpi.com/article/10.3390/biom15030398/s1, Table S1. Donor ratios per NOC. (gray = degraded donor); Table S2. Analytical thresholds; Table S3. Exclusionary and inclusionary LRs with NOC+1; Table S4. Percentage of adventitious matches in which the true donors resulted in larger LRs than the non-donor relative; Table S5. eNOC sub-source LRS organized by SWGDAM verbal qualifiers; Table S6. aNOC sub-source LRS organized by SWGDAM verbal qualifiers; Figure S1. Comparison of adventitious log (LRs) with aNOC vs. eNOC. (a) aNOC = 1. (b) aNOC = 2. (c) aNOC = 3. (d) aNOC = 4. (e) aNOC = 5; Figure S2. Zoomed-in comparison of adventitious log (LRs) with aNOC vs. eNOC. (a) aNOC=1. (b) aNOC = 2. (c) aNOC = 3. (d) aNOC = 4. (e) aNOC = 5. Light blue: eNOC=1; orange: eNOC = 2; gray: eNOC = 3; yellow: eNOC = 4; dark blue: eNOC = 5; Figure S3. Familial search log (LRs) > 0 (eNOC = 3 STRmix^TM^ v2.7 with GeneMapper^TM^ data) blue triangle: known donor; orange asterisk: simulated non-donor parents/children; black x: unrelated non-donors.
