# Supplementary material for: Assessing Adventitious Matches of Non-Donors Related to True Contributors
Source: Biomolecules. 2025 Mar 11;15(3):398. doi: 10.3390/biom15030398 (PMC11940124; doi:10.3390/biom15030398)
Supplement: Supplementary file 1 [file biomolecules-15-00398-s001.zip › biomolecules-3293544-supplementary.pdf]

Supplementary

Table S1. Donor ratios per NOC. (gray= degraded donor)

2-person			3-person				4-person					5-person					
Mx1-Mx10	0.01	0.99	Mx241-Mx250	0.01	0.01	0.98	Mx481-Mx490	0.01	0.01	0.01	0.97	Mx721-Mx730	0.01	0.01	0.01	0.01	0.96
Mx11-Mx20	0.03	0.97	Mx251-Mx260	0.03	0.03	0.94	Mx491-Mx500	0.03	0.03	0.03	0.91	Mx731-Mx740	0.03	0.03	0.03	0.03	0.88
Mx21-Mx30	0.05	0.95	Mx261-Mx270	0.05	0.05	0.9	Mx501-Mx510	0.05	0.05	0.05	0.85	Mx741-Mx750	0.05	0.05	0.05	0.05	0.8
Mx31-Mx40	0.1	0.9	Mx271-Mx280	0.1	0.1	0.8	Mx511-Mx520	0.1	0.1	0.1	0.7	Mx751-Mx760	0.1	0.1	0.1	0.1	0.6
Mx41-Mx50	0.2	0.8	Mx281-Mx290	0.2	0.2	0.6	Mx521-Mx530	0.2	0.2	0.2	0.4	Mx761-Mx770	0.15	0.15	0.15	0.15	0.4
Mx51-Mx60	0.3	0.7	Mx291-Mx300	0.3	0.3	0.4	Mx531-Mx540	0.01	0.01	0.49	0.49	Mx771-Mx780	0.01	0.01	0.01	0.49	0.49
Mx61-Mx70	0.4	0.6	Mx301-Mx310	0.01	0.49	0.5	M541-Mx550	0.03	0.01	0.48	0.48	Mx781-Mx790	0.03	0.03	0.03	0.46	0.46
Mx71-Mx80	0.5	0.5	Mx311-Mx320	0.03	0.48	0.49	Mx551-Mx560	0.05	0.01	0.47	0.47	Mx791-Mx800	0.05	0.05	0.05	0.43	0.43
Mx81-Mx90	0.01	0.99	Mx321-Mx330	0.05	0.47	0.48	Mx561-Mx570	0.1	0.1	0.4	0.4	Mx801-Mx810	0.1	0.1	0.1	0.35	0.35
Mx91-Mx100	0.03	0.97	Mx331-Mx340	0.1	0.45	0.45	Mx571-Mx580	0.2	0.2	0.3	0.3	Mx811-Mx820	0.01	0.01	0.33	0.33	0.33
Mx101-Mx110	0.05	0.95	Mx341-Mx350	0.2	0.4	0.4	Mx581-Mx590	0.01	0.33	0.33	0.33	Mx821-Mx830	0.03	0.03	0.31	0.31	0.31
Mx111-Mx120	0.1	0.9	Mx351-Mx360	0.3	0.35	0.35	Mx591-Mx600	0.03	0.32	0.32	0.33	Mx831-Mx840	0.05	0.05	0.3	0.3	0.3
Mx121-Mx130	0.2	0.8	Mx361-Mx370	0.01	0.33	0.66	Mx601-Mx610	0.05	0.31	0.31	0.33	Mx841-Mx850	0.1	0.1	0.27	0.27	0.27
Mx131-Mx140	0.3	0.7	Mx371-Mx380	0.03	0.32	0.65	Mx611-Mx620	0.1	0.3	0.3	0.3	Mx851-Mx860	0.01	0.24	0.25	0.25	0.25
Mx141-Mx150	0.4	0.6	Mx381-Mx390	0.05	0.31	0.64	Mx621-Mx630	0.2	0.27	0.27	0.27	Mx861-Mx870	0.03	0.24	0.24	0.24	0.25

Mx151-Mx160	0.5	0.5	Mx391-Mx400	0.1	0.3	0.6	Mx631-Mx640	0.25	0.25	0.25	0.25	Mx871-Mx880	0.05	0.23	0.24	0.24	0.24
Mx161-Mx170	0.01	0.99	Mx401-Mx410	0.2	0.26	0.54	Mx641-M650	0.21	0.23	0.27	0.29	Mx881-Mx890	0.1	0.22	0.22	0.23	0.23
Mx171-Mx180	0.03	0.97	Mx411-Mx420	0.01	0.25	0.74	Mx651-Mx660	0.15	0.2	0.3	0.35	Mx891-Mx900	0.2	0.2	0.2	0.2	0.2
Mx181-Mx190	0.05	0.95	Mx421-Mx430	0.03	0.24	0.73	Mx661-Mx670	0.05	0.15	0.35	0.45	Mx901-Mx910	0.14	0.17	0.2	0.23	0.26
Mx191-Mx200	0.1	0.9	Mx431-Mx440	0.05	0.24	0.71	Mx671-Mx680	0.01	0.14	0.35	0.5	Mx911-Mx920	0.01	0.09	0.2	0.3	0.4
Mx201-Mx210	0.2	0.8	Mx441-Mx450	0.1	0.23	0.68	Mx681-Mx690	0.1	0.1	0.3	0.5	Mx921-Mx930	0.01	0.01	0.1	0.44	0.44
Mx211-Mx220	0.3	0.7	Mx451-Mx460	0.33	0.33	0.33	Mx691-Mx700	0.1	0.2	0.2	0.5	Mx931-Mx940	0.05	0.05	0.15	0.37	0.38
Mx221-Mx230	0.4	0.6	Mx461-Mx470	0.33	0.33	0.33	Mx701-Mx710	0.05	0.05	0.1	0.8	Mx941-Mx950	0.1	0.1	0.2	0.3	0.3
Mx231-Mx240	0.5	0.5	Mx471-Mx480	0.33	0.33	0.33	Mx711-Mx720	0.05	0.05	0.2	0.7	Mx951-Mx960	0.05	0.05	0.23	0.23	0.44

Table S2. Analytical Thresholds

**Dye**	**GeneMapper (RFU)**	**FaSTR (RFU)**
Blue	55	30
Green	80	50
Yellow	40	30
Red	55	50
Purple	65	50

Table S3. Exclusionary and Inclusionary LRs with NOC+1

**NOC$_{(aNOC=eNOC)}$+1**		**2p+1**	**3p+1**	**4p+1**
Sibling	LR<1	49%	67%	69%
	LR>1	51%	33%	31%
Parent/ Child	LR<1	58%	73%	74%
	LR>1	42%	27%	26%
Unrelated	LR<1	94%	95%	98%
	LR>1	6%	5%	2%

Table S4. Percentage of adventitious matches in which the true donor resulted in larger LRs than the non-donor relative.

**aNOC**	**2**	**3**	**4**	**5**
Sibling	99%	98%	98%	96%
Parent/ Child	98%	99%	98%	96%

Table S5. eNOC sub-source LRS organized by SWGDAM verbal qualifiers.

		Simulated Sibling					Simulated Parent/ Child					Unrelated				
eNOC	Total conc.	Excluded	Limited Support	Moderate Support	Strong Support	Very Strong	Excluded	Limited Support	Moderate Support	Strong Support	Very Strong	Excluded	Limited Support	Moderate Support	Strong Support	Very Strong
1	125	99.88%	0.13%	0.00%	0.00%	0.00%	100.00%	0.00%	0.00%	0.00%	0.00%	100.00%	0.00%	0.00%	0.00%	0.00%
	62	100.00%	0.00%	0.00%	0.00%	0.00%	100.00%	0.00%	0.00%	0.00%	0.00%	99.88%	0.03%	0.09%	0.00%	0.00%
	31	97.00%	1.63%	1.00%	0.25%	0.13%	98.25%	1.50%	0.13%	0.13%	0.00%	100.00%	0.00%	0.00%	0.00%	0.00%
	16	87.87%	7.73%	3.20%	0.93%	0.27%	94.27%	3.33%	2.27%	0.13%	0.00%	99.78%	0.22%	0.00%	0.00%	0.00%
	8	66.40%	27.20%	5.07%	1.33%	0.00%	68.67%	26.53%	3.87%	0.93%	0.00%	94.04%	5.90%	0.06%	0.00%	0.00%
2	4000	99.33%	0.39%	0.22%	0.06%	0.00%	99.44%	0.38%	0.19%	0.00%	0.00%	100.00%	0.00%	0.00%	0.00%	0.00%
	2000	98.65%	0.78%	0.35%	0.22%	0.00%	99.00%	0.74%	0.22%	0.04%	0.00%	99.86%	0.14%	0.00%	0.00%	0.00%
	1000	97.41%	2.14%	0.45%	0.00%	0.00%	97.18%	2.41%	0.41%	0.00%	0.00%	99.58%	0.42%	0.00%	0.00%	0.00%
	500	94.63%	4.67%	0.58%	0.08%	0.04%	95.13%	4.17%	0.54%	0.17%	0.00%	99.17%	0.83%	0.00%	0.00%	0.00%
	250	94.33%	4.71%	0.96%	0.00%	0.00%	94.21%	5.00%	0.71%	0.08%	0.00%	98.42%	1.58%	0.00%	0.00%	0.00%
	125	90.79%	7.79%	1.04%	0.33%	0.04%	90.88%	8.38%	0.50%	0.13%	0.13%	97.94%	2.06%	0.00%	0.00%	0.00%
	62	72.17%	14.83%	8.88%	3.38%	0.75%	74.17%	16.83%	7.96%	0.92%	0.13%	98.34%	1.66%	0.00%	0.00%	0.00%
	31	52.79%	26.50%	16.21%	3.33%	1.17%	50.42%	33.13%	14.13%	2.21%	0.13%	97.49%	2.51%	0.00%	0.00%	0.00%
	16	48.71%	41.00%	7.79%	2.21%	0.29%	47.96%	43.54%	7.25%	1.17%	0.08%	92.53%	7.45%	0.02%	0.00%	0.00%
	8	60.87%	34.17%	4.48%	0.43%	0.04%	80.16%	15.56%	3.71%	0.53%	0.05%	91.06%	8.92%	0.02%	0.00%	0.00%
3	4000	99.11%	0.53%	0.19%	0.11%	0.06%	99.69%	0.25%	0.06%	0.00%	0.00%	100.00%	0.00%	0.00%	0.00%	0.00%
	2000	96.69%	2.00%	1.03%	0.22%	0.06%	97.14%	1.83%	0.89%	0.14%	0.00%	99.96%	0.04%	0.00%	0.00%	0.00%
	1000	96.81%	2.28%	0.61%	0.25%	0.06%	97.64%	1.67%	0.58%	0.08%	0.03%	99.88%	0.12%	0.00%	0.00%	0.00%
	500	90.81%	6.89%	1.72%	0.44%	0.14%	92.03%	6.61%	1.25%	0.11%	0.00%	99.46%	0.54%	0.00%	0.00%	0.00%
	250	86.99%	9.65%	2.61%	0.55%	0.20%	88.70%	9.39%	1.86%	0.06%	0.00%	99.01%	0.97%	0.02%	0.00%	0.00%
	125	78.48%	16.27%	4.33%	0.58%	0.33%	80.45%	16.82%	2.45%	0.21%	0.06%	98.71%	1.29%	0.00%	0.00%	0.00%
	62	56.58%	31.11%	9.56%	2.31%	0.44%	58.03%	32.67%	8.14%	1.14%	0.03%	98.02%	1.98%	0.00%	0.00%	0.00%
	31	53.94%	32.56%	10.72%	2.50%	0.28%	54.75%	33.28%	10.58%	1.28%	0.11%	97.50%	2.50%	0.00%	0.00%	0.00%
	16	55.16%	37.91%	6.00%	0.84%	0.09%	54.20%	39.68%	5.59%	0.46%	0.06%	92.90%	7.10%	0.00%	0.00%	0.00%
	8	58.61%	38.75%	2.61%	0.03%	0.00%	58.17%	39.50%	2.33%	0.00%	0.00%	86.26%	13.74%	0.00%	0.00%	0.00%
4	4000	92.78%	4.83%	1.90%	0.30%	0.20%	95.23%	4.05%	0.68%	0.05%	0.00%	99.78%	0.21%	0.02%	0.00%	0.00%
	2000	90.23%	6.90%	2.29%	0.54%	0.04%	92.90%	5.85%	1.08%	0.15%	0.02%	99.63%	0.37%	0.00%	0.00%	0.00%
	1000	87.96%	9.93%	1.43%	0.50%	0.17%	91.28%	7.51%	1.11%	0.10%	0.01%	99.09%	0.91%	0.00%	0.00%	0.00%
	500	86.89%	10.85%	1.76%	0.43%	0.07%	90.00%	8.78%	1.14%	0.08%	0.01%	98.76%	1.24%	0.00%	0.00%	0.00%
	250	84.54%	12.75%	2.21%	0.31%	0.19%	86.98%	11.96%	1.00%	0.06%	0.00%	98.09%	1.91%	0.00%	0.00%	0.00%
	125	71.80%	22.63%	4.76%	0.74%	0.07%	74.72%	22.15%	2.93%	0.17%	0.02%	97.99%	1.99%	0.02%	0.00%	0.00%
	62	56.98%	32.08%	9.06%	1.71%	0.17%	57.77%	34.08%	7.13%	0.96%	0.06%	97.82%	2.18%	0.00%	0.00%	0.00%
	31	55.72%	35.65%	7.63%	1.00%	0.00%	55.78%	36.96%	6.80%	0.46%	0.00%	95.41%	4.59%	0.00%	0.00%	0.00%
	16	58.44%	36.17%	4.33%	0.85%	0.21%	58.77%	36.08%	4.56%	0.56%	0.02%	93.13%	6.85%	0.02%	0.00%	0.00%
	8	59.98%	38.25%	1.63%	0.15%	0.00%	59.31%	39.19%	1.46%	0.04%	0.00%	86.08%	13.90%	0.02%	0.00%	0.00%
5	2000	80.10%	18.00%	1.80%	0.10%	0.00%	84.15%	14.90%	0.90%	0.05%	0.00%	98.30%	1.70%	0.00%	0.00%	0.00%
	1000	81.71%	15.73%	2.17%	0.36%	0.02%	84.80%	14.17%	0.97%	0.04%	0.02%	98.49%	1.51%	0.00%	0.00%	0.00%
	500	79.64%	17.00%	2.87%	0.42%	0.04%	82.51%	15.87%	1.49%	0.13%	0.00%	98.06%	1.92%	0.02%	0.00%	0.00%
	250	71.57%	25.46%	2.61%	0.33%	0.03%	73.83%	24.24%	1.88%	0.05%	0.00%	97.87%	2.07%	0.06%	0.00%	0.00%
	125	60.57%	34.86%	4.06%	0.50%	0.02%	63.33%	33.35%	3.16%	0.15%	0.00%	96.40%	3.60%	0.00%	0.00%	0.00%
	62	59.02%	33.33%	6.62%	1.02%	0.02%	58.55%	35.80%	5.27%	0.38%	0.00%	95.44%	4.54%	0.02%	0.00%	0.00%
	31	61.01%	33.34%	5.25%	0.40%	0.00%	59.65%	35.70%	4.47%	0.17%	0.00%	93.86%	6.12%	0.02%	0.00%	0.00%
	16	60.20%	37.07%	2.58%	0.13%	0.02%	58.20%	39.22%	2.50%	0.08%	0.00%	88.85%	11.13%	0.02%	0.00%	0.00%
	8	60.43%	39.22%	0.35%	0.00%	0.00%	60.10%	39.60%	0.30%	0.00%	0.00%	78.58%	21.40%	0.02%	0.00%	0.00%

Table S6. aNOC sub-source LRS organized by SWGDAM verbal qualifiers.

		Simulated Sibling					Simulated Parent/ Child					Unrelated				
aNOC	Total conc.	Excluded	Limited Support	Moderate Support	Strong Support	Very Strong Support	Excluded	Limited Support	Moderate Support	Strong Support	Very Strong Support	Excluded	Limited Support	Moderate Support	Strong Support	Very Strong Support
1	125	99.89%	0.11%	0.00%	0.00%	0.00%	100.00%	0.00%	0.00%	0.00%	0.00%	99.98%	0.00%	0.02%	0.00%	0.00%
	62	99.63%	0.07%	0.30%	0.00%	0.00%	100.00%	0.00%	0.00%	0.00%	0.00%	99.90%	0.04%	0.06%	0.00%	0.00%
	31	98.50%	0.78%	0.50%	0.11%	0.11%	99.22%	0.67%	0.06%	0.06%	0.00%	99.98%	0.00%	0.00%	0.02%	0.00%
	16	96.73%	2.08%	0.77%	0.32%	0.10%	97.88%	1.29%	0.65%	0.14%	0.04%	99.90%	0.07%	0.02%	0.00%	0.02%
	8	96.03%	2.96%	0.85%	0.15%	0.01%	96.08%	2.97%	0.85%	0.10%	0.00%	98.81%	1.10%	0.06%	0.02%	0.005%
2	4000	99.29%	0.41%	0.24%	0.06%	0.00%	99.47%	0.35%	0.18%	0.00%	0.00%	100.00%	0.00%	0.00%	0.00%	0.00%
	2000	98.59%	0.82%	0.36%	0.23%	0.00%	99.38%	0.48%	0.10%	0.05%	0.00%	99.85%	0.15%	0.00%	0.00%	0.00%
	1000	97.57%	2.00%	0.43%	0.00%	0.00%	97.36%	2.26%	0.38%	0.00%	0.00%	99.58%	0.42%	0.00%	0.00%	0.00%
	500	96.27%	3.06%	0.55%	0.08%	0.04%	96.08%	3.25%	0.51%	0.16%	0.00%	99.40%	0.60%	0.00%	0.00%	0.00%
	250	94.57%	4.57%	0.86%	0.00%	0.00%	94.62%	4.69%	0.62%	0.07%	0.00%	98.49%	1.50%	0.02%	0.00%	0.00%
	125	93.09%	5.44%	1.12%	0.33%	0.02%	93.19%	5.95%	0.65%	0.14%	0.07%	98.48%	1.52%	0.00%	0.00%	0.00%
	62	89.14%	6.44%	2.72%	1.35%	0.35%	90.41%	6.52%	2.48%	0.53%	0.06%	99.51%	0.49%	0.00%	0.00%	0.00%
	31	86.65%	8.31%	3.95%	0.88%	0.21%	86.55%	9.41%	3.47%	0.55%	0.03%	99.34%	0.66%	0.00%	0.00%	0.00%
	16	77.70%	17.82%	3.74%	0.61%	0.13%	77.88%	18.38%	3.38%	0.33%	0.04%	97.60%	2.39%	0.02%	0.00%	0.00%
	8	75.63%	22.09%	2.02%	0.21%	0.05%	76.00%	21.91%	2.00%	0.09%	0.00%	92.72%	7.17%	0.11%	0.00%	0.00%
3	4000	99.03%	0.62%	0.21%	0.10%	0.05%	99.54%	0.41%	0.05%	0.00%	0.00%	99.91%	0.09%	0.00%	0.00%	0.00%
	2000	94.67%	4.03%	1.06%	0.20%	0.05%	96.02%	3.01%	0.84%	0.12%	0.00%	99.70%	0.30%	0.00%	0.00%	0.00%
	1000	96.24%	2.70%	0.67%	0.33%	0.07%	97.20%	1.85%	0.87%	0.07%	0.02%	99.73%	0.27%	0.00%	0.00%	0.00%
	500	92.20%	5.71%	1.60%	0.37%	0.11%	93.08%	5.63%	1.21%	0.09%	0.00%	99.63%	0.37%	0.00%	0.00%	0.00%
	250	88.80%	8.00%	2.61%	0.39%	0.20%	90.34%	8.04%	1.59%	0.04%	0.00%	99.44%	0.56%	0.00%	0.00%	0.00%
	125	89.77%	7.37%	2.23%	0.41%	0.21%	92.02%	6.40%	1.37%	0.18%	0.03%	99.51%	0.49%	0.00%	0.00%	0.00%
	62	83.18%	11.42%	4.42%	0.91%	0.08%	85.06%	11.30%	3.18%	0.42%	0.04%	99.77%	0.23%	0.00%	0.00%	0.00%
	31	74.52%	19.93%	4.78%	0.78%	0.00%	75.48%	19.00%	5.00%	0.52%	0.00%	99.10%	0.86%	0.04%	0.00%	0.00%
	16	66.83%	31.67%	1.50%	0.00%	0.00%	71.67%	27.00%	1.17%	0.17%	0.00%	95.64%	4.36%	0.00%	0.00%	0.00%
	8															
4	4000	93.06%	4.81%	1.69%	0.27%	0.18%	94.57%	4.62%	0.79%	0.02%	0.00%	99.73%	0.25%	0.02%	0.00%	0.00%
	2000	92.82%	4.87%	1.83%	0.42%	0.05%	94.35%	4.63%	0.90%	0.10%	0.02%	99.77%	0.23%	0.00%	0.00%	0.00%
	1000	92.32%	6.14%	1.05%	0.36%	0.13%	93.82%	5.57%	0.54%	0.07%	0.00%	99.45%	0.55%	0.00%	0.00%	0.00%
	500	90.42%	7.55%	1.61%	0.38%	0.05%	91.97%	6.95%	1.00%	0.06%	0.02%	99.52%	0.48%	0.00%	0.00%	0.00%
	250	90.33%	7.49%	1.77%	0.33%	0.08%	92.59%	6.48%	0.88%	0.05%	0.00%	99.66%	0.34%	0.00%	0.00%	0.00%
	125	79.93%	15.65%	3.73%	0.69%	0.00%	82.25%	14.64%	2.91%	0.17%	0.02%	99.77%	0.23%	0.00%	0.00%	0.00%
	62	74.20%	20.20%	4.20%	1.40%	0.00%	78.24%	17.17%	4.19%	0.40%	0.00%	99.53%	0.47%	0.00%	0.00%	0.00%
	31															
	16															
	8															
5	2000	76.69%	20.76%	2.41%	0.14%	0.00%	83.45%	15.24%	1.17%	0.14%	0.00%	98.20%	1.80%	0.00%	0.00%	0.00%
	1000	83.43%	13.34%	2.74%	0.46%	0.03%	86.46%	12.37%	1.11%	0.03%	0.03%	99.16%	0.84%	0.00%	0.00%	0.00%
	500	80.00%	15.28%	3.95%	0.72%	0.05%	84.26%	13.74%	1.90%	0.10%	0.00%	98.94%	1.00%	0.06%	0.00%	0.00%
	250	72.00%	25.68%	1.89%	0.32%	0.11%	76.74%	22.00%	1.16%	0.11%	0.00%	98.71%	1.17%	0.12%	0.00%	0.00%
	125	56.80%	42.00%	0.80%	0.40%	0.00%	57.60%	40.40%	1.60%	0.40%	0.00%	98.59%	1.41%	0.00%	0.00%	0.00%
	62															
	31															
	16															
	8															

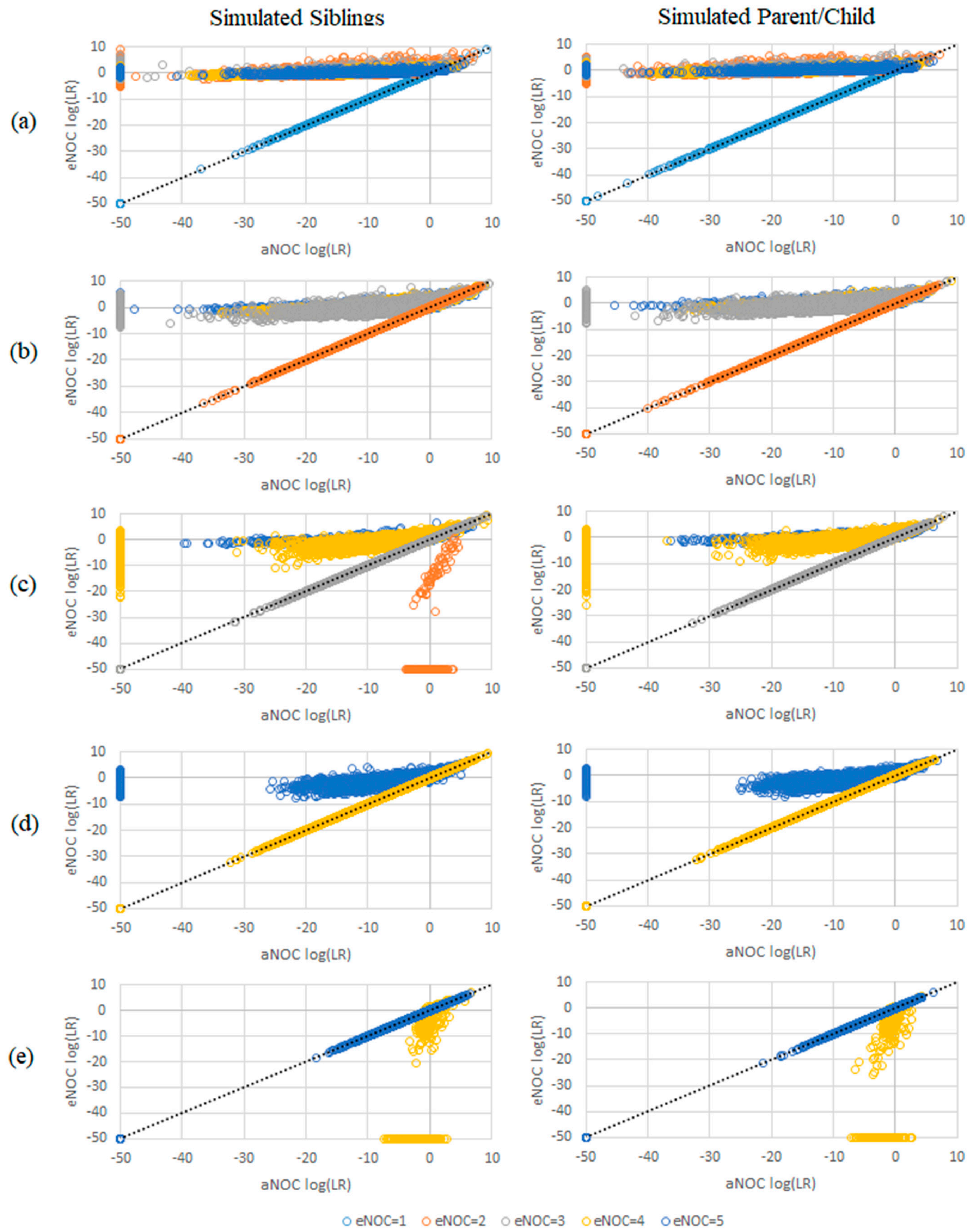

Figure S1. Comparison of adventitious log(LR)s with aNOC vs. eNOC. (a) aNOC=1. (b) aNOC=2. (c) aNOC=3. (d) aNOC=4. (e) aNOC=5.

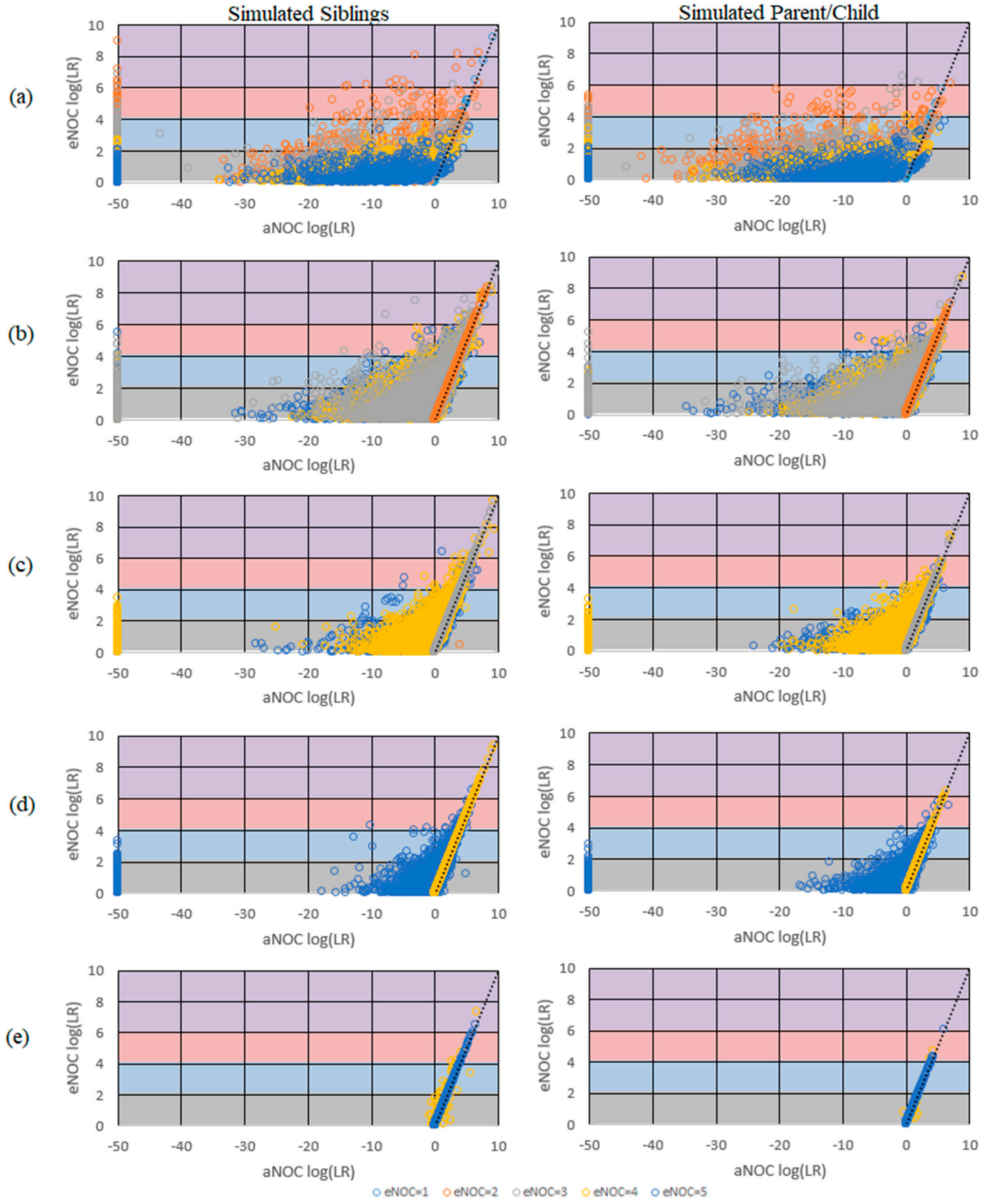

Figure S2. Zoomed in comparison of adventitious log(LR)s with aNOC vs. eNOC. (a) aNOC=1. (b) aNOC=2. (c) aNOC=3. (d) aNOC=4. (e) aNOC=5. Light blue: eNOC=1; orange: eNOC=2; grey: eNOC=3; yellow: eNOC=4; dark blue: eNOC=5.

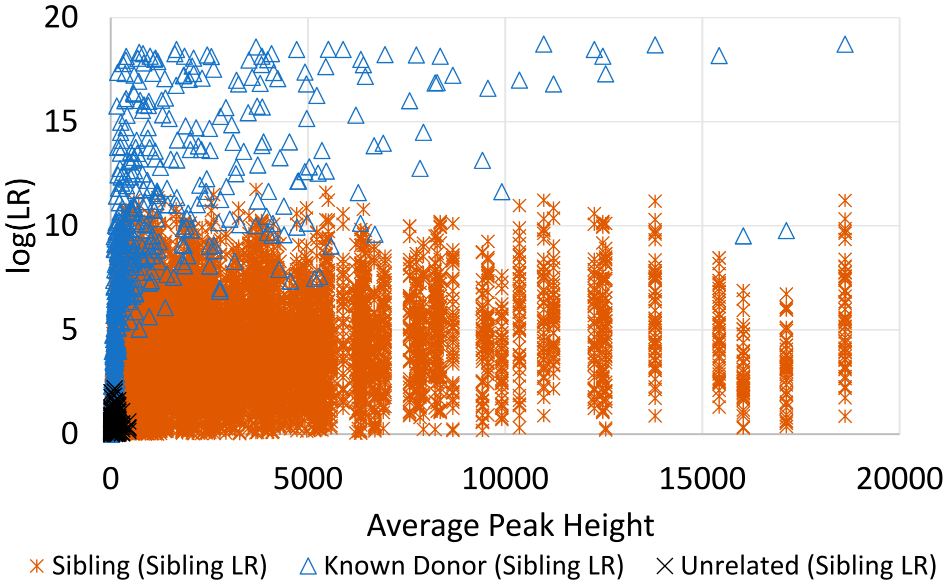

Figure S3. Familial Search log(LRs)>0 (eNOC=3 STRmix™ v2.7 with GeneMapper™ data) blue triangle: known donor; orange asterisk: simulated non-donor parent/children; black x: unrelated non-donors.
